# Equivalent hip stem fixation by Hi-Fatigue G and Palacos R + G bone cement: a randomized radiostereometric controlled trial of 52 patients with 2 years’ follow-up

**DOI:** 10.1080/17453674.2019.1595390

**Published:** 2019-04-01

**Authors:** Peter B Jørgensen, Martin Lamm, Kjeld Søballe, Maiken Stilling

**Affiliations:** a Department of Orthopedic Surgery, Aarhus University Hospital;;; b Department of Clinical Medicine, Aarhus University, Denmark

## Abstract

Background and purpose — Long-term fixation of cemented femoral stems relies on several factors including cement adhesion and fatigue. Hi-Fatigue is a newer third-generation bone cement with low-viscosity properties at room temperature, good mechanical strength, and stable bone–cement interface in a laboratory testing environment. Palacos bone cement has excellent 10-year survival and is considered gold standard. We compared stem subsidence after fixation with Hi-Fatigue and Palacos bone cements using radiostereometry.

Patients and methods — In a patient-blinded randomized controlled trial, 52 patients (30 women) at mean age 76 years (71–87) with osteoarthrosis and no osteoporosis received Hi-Fatigue G or Palacos R + G cement fixation of collarless, polished, double-tapered stems (CPT). Tantalum beads were inserted in the periprosthetic bone. Supine stereoradiographs were obtained postoperatively, 3 months, 6 months, 1 year, and 2 years after surgery. Oxford Hip Score (OHS) and VAS pain were recorded preoperatively and 1 and 2 years after surgery. Cement working times and properties were registered.

Results — At 2 years, mean stem subsidence of 1.12 mm (95% CI 0.96–1.29) for Hi-Fatigue and 1.19 mm (CI 1.03–1.34) for Palacos was similar. Likewise, stem version was comparable between cement groups. Mean OHS and VAS pain were similar between cement groups.

Cement working times were similar between cement groups, but the mean curing time was longer for Hi-Fatigue (13.7 min) than for Palacos (11.6 min).

Interpretation — We found similar and generally low migration of CPT femoral stems inserted with Hi-Fatigue and Palacos bone cement until 2 years’ follow-up, which indicates a good long-term survival of polished taper femoral stems inserted with both cement types.

Palacos bone cement was introduced in the early 1970s, is widely used, and has a reported 10-year implant survival rate between 92.6% and 98.8%, when used in hip arthroplasties (Junnila et al. 2016), and may be considered a gold standard bone cement (van der Voort et al. 2015, Junnila et al. 2016). Hi-Fatigue G bone cement is a newer third-generation bone cement, with a low initial viscosity but without long-term follow-up data.

Both Hi-Fatigue G and Palacos R + G bone cements surpass international standards for stability tests according to ISO 5833:2002 (ISO 2002). They both contain gentamicin sulphate (Hi-Fatigue G 0.55/40 g, Palacos 0.8/40 g) and are sterilized using ethylene oxide. The radiopaque medium for both cements is zirconium dioxide, but the concentration is lower in Hi-Fatigue G (12%) compared with Palacos (17%), which acts positively for the mechanical stability of Hi-Fatigue G bone cement (Arora et al. 2013). Compared with Palacos R + G, Hi-Fatigue G bone cement has a lower initial viscosity that allow for good cement-to-bone penetration, interface strength, and a better fatigue life, minimizing the risk of cement failure (Rey et al. 1987, Stone et al. 1996, Race et al. 2006, Tanner 2008). These features may lead to better implant fixation with Hi-Fatigue G bone cement compared with Palacos R + G.

RSA can be used to measure implant migration with respect to tantalum markers in the surrounding bone as part of a phased introduction (Nelissen et al. 2011). Stem subsidence and retroversion have been shown to be good predictors of implant survival and this has further been used and suggested as a standard in the evaluation of new bone cements (Karrholm et al. 1994, Hauptfleisch et al. 2006). We hypothesized less subsidence of polished femoral stems fixed with Hi-Fatigue G bone cement compared with polished femoral stems fixed with Palacos bone cement.

## Patients and methods

The study design was a patient-blinded, randomized, controlled study.

**Figure 1.**
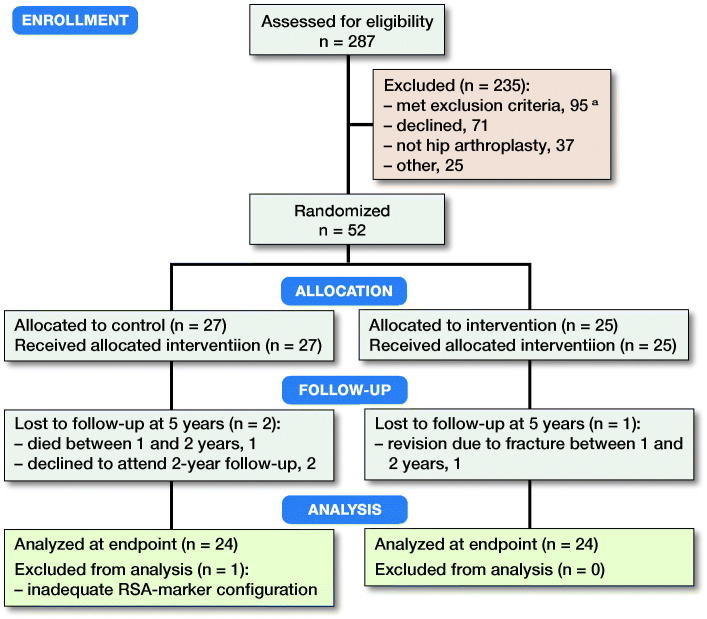
Consort flow chart. **^a^**In 2012 patients taking medication with vitamin K antagonists were removed from the list of contraindications, because its relevance disappeared with increased use of NOAK. Until then 16 patients were excluded due to use of vitamin K antagonists.

Between November 2010 and March 2014, 52 patients (30 women) with a mean age of 76 years (71–87) were included in the study (Figure 1). The criteria for inclusion were primary osteoarthritis, age 71 years and above, preoperative T-score above –2.5 (meaning no osteoporosis), and informed consent. The exclusion criteria were neuromuscular and vascular disease, former proximal femoral fracture, osteonecrosis of the femoral head, pharmacological (NSAID, estrogens, cortisone), metabolic bone disease, senile dementia, alcohol or drug abuse, major psychiatric disease, metastatic cancer/radiation or chemotherapy, poor dental status (infection risk), spine disease, or severe systemic disease (i.e. hemiparesis or Parkinson’s disease). Sample size calculation indicated 24 patients per group based on a clinically relevant difference in subsidence of 0.33 mm (SD 0.39) with a power of 90% and alpha set to 0.05 (Karrholm et al. 1994, Glyn-Jones et al. 2006). To balance postoperative dropout, we aimed for 25 patients per group. To compensate for dropouts during the inclusion period of the study we included an additional 2 patients.

Randomization was done within 6 blocks of 10 patients (5 CPT stems fixed with Hi-Fatigue G bone cement, and 5 CPT stems fixed with Palacos R + G bone cement) by drawing concealed labels from sequentially numbered closed envelopes. Randomization was done in theater. The surgeon was not blinded to the type of cement used. 27 patients received stem fixation by Hi-Fatigue G bone cement (Zimmer Biomet) and 25 patients received stem fixation by Palacos R + G bone cement (Heraeus).

The components used were the collarless, polished, double-tapered CPT (12–14 conus Cr-Co) femoral stem (Zimmer Biomet), which has shown excellent long-term results (DHR 2016), the cementless Trilogy Fiber-Mesh Cup (Zimmer Biomet) with optional screw fixation and a highly crosslinked Longevity polyethylene liner (Zimmer Biomet). Femoral heads were CoCr size 36 mm.

The cement was stored in the theater at 20° C (18.3–21.5) and at 44% humidity (17–78) for at least 24 hours before surgeries, and in similar general storage conditions with temperature 20° C (17.5– 21.4) and at 48% humidity (24–78) with some seasonal variation. Both types of bone cement were vacuum mixed with the closed MixiGun system (Zimmer Biomet). The cement curing time was monitored using a digital timer with 1-second resolution. Nurses and surgeons also evaluated the consistency and user friendliness of each bone cement type on a numeric scale from 1 to 9.

All procedures were performed by 6 experienced hip surgeons. A preoperative plan was made using standardized digital radiographs with a 30 mm metal ball marker, at the level of the greater trochanter, and the AGFA OT3000 digital templating software [AGFA, Vancouver], for optimal correction of lateral and vertical offsets, and optimal postoperative leg length. A posterolateral approach was used. During surgery 1-mm tantalum beads were inserted into the peri-prosthetic femoral bone (lesser and greater trochanter). Peroperative leg length and stability was assessed before and after implantation using trial components (the femoral rasp, and trial heads). All patients received prophylactic antibiotics: intravenous cefuroxime 1.5 g preoperatively and 1.5 g 3 times in the postoperative 24 hours, and thrombo-prophylactic treatment: subcutaneous Arixtra (fondaparinux) 2.5 mg/day or Innohep (tinzaparin) 4,500 anti-Xa IE/day postoperatively until discharge.

Postoperatively the patient was mobilized with full weight-bearing and walking aids as needed, using a “fast track” protocol.

### Pre- and postoperative characteristics of the study population (Table 1)

**Figure 2.**
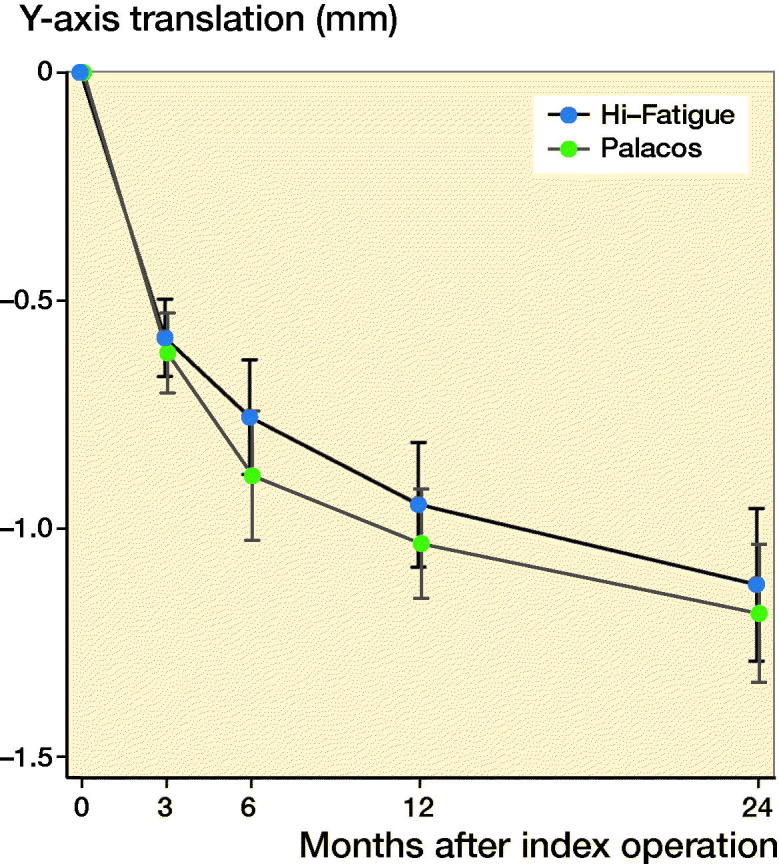
Oxford Hip Scores: preoperative, 12, and 24 months postoperatively. Both Hi-Fatigue G (p < 0.001) and Palacos R + G (p < 0.001) achieved significant increase from 0 to 12 months with no statistically significant difference between groups (p = 0.3).

Patients assessed for study participation and follow-up of randomized participants are shown in the CONSORT flowchart (Figure 1). 1 patient was excluded during surgery because the MixiGun jammed twice during application of cement, due to a human error. At 2 years’ follow-up, there have been no revisions due to aseptic implant loosening. 1 patient suffered a traumatic periprosthetic fracture 18 months after operation and received revision of the stem and osteosynthesis of the fracture. 2 patients suffered hip dislocation within the first 3 months, 1 of these combined with avulsion of the greater trochanter. Another patient had avulsion of the greater trochanter post-surgery without known trauma. Both avulsions were treated nonoperatively. There was 1 periprosthetic infection 1 month postoperatively treated with soft tissue debridement and change of acetabular liner and metal head. This patient had a full recovery but died of causes unrelated to the periprosthetic infection 2 months before 2-year follow-up. The clinical scores (OHS and VAS pain) were similar at 1-year follow-up and 2-year follow-up between groups (Figure 2). At 2-year follow-up, VAS pain and OHS correlated neither with subsidence (rho < 0.2) nor with retroversion (rho < 0.01) (Table 2, see Supplementary data).

**Table 1.** Baseline demographics. Values are mean (95% CI) or frequency

Baseline demographics	Hi-Fatigue	Palacos
n	25	27
Age	76 (74–78)	76 (75–78)
Sex (male/female), n	13/12	9/18
T-score	–0.9 (–1.3 to –0.5)	–1.1 (–1.5 to –0.8)
BMI	29 (27–31)	29 (27–31)
Oxford Hip Score	25 (20–28)	22 (19–25)
Pain rest	3.5 (2.2–4.8)	3.4 (2.4–4.4)
Pain activity	5.7 (4.5–6.8)	6.3 (5.4–7.1)

Oxford Hip Score: 0–48, 48 being best

### Primary outcome measure: RSA

A standard RSA setup of 2 synchronized ceiling-fixed roentgen tubes (Arco-Ceil/Medira; Santax Medico, Aarhus, Denmark) angled toward each other at 40° were used. All radiographs were fully digital (Fuji CR, image size 35 x 43) and were stored in DICOM file format without compression. During this study the RSA equipment was replaced with a newer direct digital dedicated stereo X-ray system, AdoraRSA suite (NRT, Aarhus, Denmark) with CXDI-70C detectors (Canon, Tokyo, Japan). The configuration remained: 2 ceiling-mounted X-ray tubes at 40 degrees angle. We used a uniplanar carbon calibration box (Box 24, Medis Specials, Leiden, Netherlands). Patients had loaded full weight on the hip prosthesis before radiostereometric examinations, which were recorded with the patient supine.

Model-Based RSA 3.34 (RSAcore, Leiden, the Netherlands) was used for RSA analysis using EGS-models (Kaptein et al. 2006). The upper limit for mean rigid body fitting error (RBE), which is the stability limit for markers used in the analysis, was per default 0.5 mm in the software. The mean rigid body error of the bone markers was 0.19 (CI 0.16–0.22). The mean condition number (dispersion of the bone markers in the femur) was 39 (CI 31–46). The difference in matching of the EGS hip-stem model to the CPT stem (model pose estimation) in the stereoradiographs was mean 0.10 mm (CI 0.01–0.11).

Double examination stereoradiographs were obtained for all patients to document the clinical precision (Valstar et al. 2005). The double examination stereoradiographs were performed with complete repositioning of the patient and the radiographic equipment between examinations. The postoperative stereoradiograph was used as the reference in the migration analysis of the double examinations, and the expected difference in displacement between the 2 calculations represents the systematic error of the RSA system (bias) and should be zero.

Based on clinical double examination stereoradiographs the precision of RSA expressed as the coefficient of repeatability (CR) was approximately 0.2 mm for translations including TT and 0.3 mm for translation on the z-axis, less than 1° for rotation about the X and Z axes, and—as expected—less precise for rotation about the Y-axis 2.1° (Table 3, see Supplementary data). 1 patient was excluded based on inadequate marker configuration with a high condition number. Another 3 patients had condition numbers between 150 and 250, which was above the suggested 150 upper limit, but the configuration of the marker models was visually good (not linear) and acceptable and RSA data were included in the study (Valstar et al. 2005, ISO 2013).

Evaluation of femoral stem migration by radiostereometric analysis was performed at 3 months, 6 months, 1 year, and 2 years with postoperative stereoradiographs as baseline.

### Secondary outcome measures

Radiographic evaluation of the cementing was performed by one blinded assessor (PBJ). Cement distribution was evaluated on the postoperative AP and Axial radiographs as either “excellent” (A), “Slight radiolucency at the cement–bone interface” (B), “Radiolucency involving 50–99% of the cement–bone interface” (C) and “Radiolucency in 100% of the cement–bone interface in any projection or failure to fil the canal such that the tip was not covered” (D), as proposed by Barrack et al. (1992).

Patient-reported outcome measures consisted of pain measured during rest and activity on a Visual Analogue Scale (VAS) from 0 to 10 (10 being the worst), and Oxford Hip Score (OHS) was between 0 and 48 (0 being worst) (Paulsen et al. 2012). VAS pain and OHS score were evaluated before operation and at 1 and 2 years after operation.

### Statistics

All continuous variables were evaluated for normality using qq-plots. The groups were then compared using Student’s t-test or non-parametric tests (Mann–Whitney U-test and Spearman’s rank correlation coefficient), as appropriate. The primary RSA endpoint was y-translation (subsidence). The secondary RSA endpoints were the remaining individual migrations along and rotations about the single axes, the summed migration in terms of total translation (TT = sqrt(Tx^2^+Ty^2^+ Tz^2^)), total rotation (TR = sqrt(Rx^2^+ Ry^2^+ Rz^2^)) and Maximum Total Point Motion (MTPM) (Selvik 1989, Valstar et al. 2005), and the clinical data. For OHS and pain scores, we present mean values and 95% confidence intervals (CI) for comparison with the literature. Statistical significance was assumed at p < 0.05. Stata/SE version 13.1 (StataCorp, College Station, TX, USA) was used for statistical computations.

### Ethics, registration, funding, and potential conflicts of interest

The study was approved by the Central Denmark Region Committee on Biomedical Research Ethics (Journal no. M-20100112; issue date: May 27, 2010) and it was registered with ClinicalTrials.gov (NCT01289834) and the Data Protection Agency (2007-58-0010, issue 19 Oct 2010). The trial was performed in compliance with the Helsinki II Declaration. The RSA analyses of this study were funded by Zimmer Biomet. All authors report no conflicts of interest.

## Results

### Primary outcome

**Figure 3.**
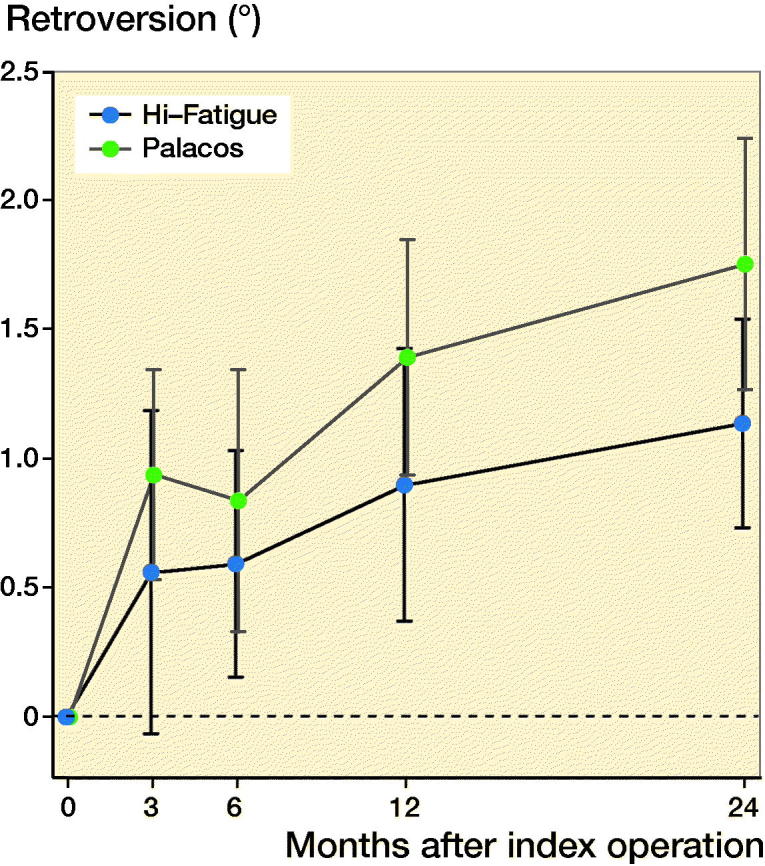
Mean subsidence (negative y-translation) of CPT stems inserted with Hi-Fatigue G and Palacos bone cements. Confidence intervals are presented in error bars, for graphical use only.

Subsidence was greater than the detection limit (0.16 mm) for all patients but not statistically significantly different for CTP stems fixed with Hi-Fatigue G bone cement and Palacos R + G bone cement respectively after both 1 year (p = 0.2) and 2 years (p = 0.7) (Figure 3, Tables 3 and 4, see Supplementary data). The estimated 2-year difference of 0.06 mm (CI –0.17 to 0.3) in subsidence between the groups excludes the clinically relevant difference of 0.33 mm. Therefore, we find the subsidence in both groups to be similar.

Likewise, all other signed translations and rotations and summed migration measures (TT, TR, MTPM) were similar between cement groups (Table 4, see Supplementary data, Figure 4).

**Figure 4.**
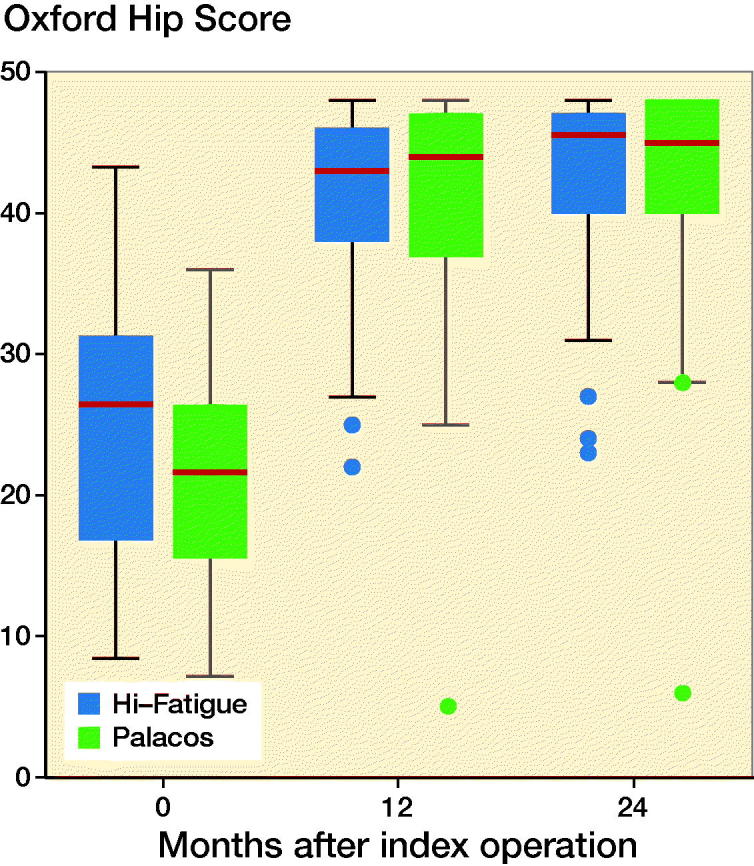
Mean y-rotation (retroversion) of CPT stems inserted with Hi-Fatigue G and Palacos bone cements. Confidence intervals are presented in error bars, for graphical use only.

At 1-year follow-up 7 patients (all Palacos R + G) surpassed the subsidence threshold of 1.2 mm, which Karrholm et al. (1994) showed to give a 50% risk of later revision in anatomic stems. At 2-year follow-up, a total of 14 patients (7 Palacos R + G) exceeded the subsidence threshold of 1.2 mm, but all had low pain scores (mean 0.8, CI 0.1–1.5) and good Oxford Hip Score (mean 45, CI 42–47).

### Secondary outcomes

Both Hi-Fatigue G and Palacos R + G showed good cement distribution (whiteout), but Palacos R + G was more often classified with slight radiolucency (n = 10) than Hi-Fatigue G (n = 1). There were no differences in stem position in the 2 groups (Table 5, see Supplementary data).

The surgical time (n = 51) was on average 85 minutes (50–150). 1 operation took a particularly long time because some equipment was contaminated and needed sterilization during the surgery, but this was not related to use of the bone cement. There were no statistically significant differences in working times for mixing and waiting for readiness to use of the bone cements (p > 0.4). Mean time of stem insertion was 16 seconds shorter for Palacos R + G (3:49) than for Hi-Fatigue G (4:05) and the mean time until total curing was 2:08 minutes longer for Hi-Fatigue G (13:46) than for Palacos R + G bone cement (11:35) (Table 6, see Supplementary data). The final curing time of Hi-Fatigue G correlated with the theater temperature and storing temperature (Table 7, see Supplementary data). No other correlations were found between mixing time, time to apply cement and stem, or total curing time and storage temperature or temperature in the theater in either of the 2 bone cements (rho < 0.24, p > 0.3).

## Discussion

The key finding of this study was similar 2-year stem subsidence and clinical results in patients operated with a collarless polished double-tapered femoral stem that was fixed with either the newer Hi-Fatigue G bone cement or Palacos R + G bone cement.

### Migration

The primary outcome of this study registered with ClinicalTrials.gov was femoral component migration in broader terms. Until now, no migration direction or pattern has been identified to clearly predict later loosening of CPT stems. However, subsidence of CPT stems has been associated with inferior cementing quality (Yates et al. 2008) and was therefore chosen in our study as the primary effect parameter. Using a single migration measure as the primary outcome also limited the risk of multiplicity testing issues.

Some subsidence is expected with the collarless polished double-tapered design, unlike the shape-closed stem designs. However, progressive and excessive migration is considered to be a proxy measure for later aseptic loosening (Nieuwenhuijse et al. 2012). In our study both bone cements provided acceptable stem fixation and the migration measures were comparable to similar RSA studies of cemented, collarless, polished, tapered femoral stems. In both cement groups the mean subsidence was more than the reported threshold of 0.33 mm (mean 0.82, CI 0.72–0.92) at 6 months, but at 2-year follow-up subsidence was less than reported threshold values that are predictive of later revision of cemented shape-closed stems (Lubinius SP1) (Karrholm et al. 1994).

The subsidence in both groups was less than 1.03 mm at 1-year follow-up, which is in accordance with other findings of 0.8 mm subsidence with the CPT stem in the first year (Yates et al. 2008). At 2-year follow-up both cement groups had subsided less than 1.2 mm. This is similar to 2-year findings of 1.36 mm of the C-stem (DePuy) (von Schewelov et al. 2014) and 1.42 mm and 0.92 mm of the Exeter stem (Stryker) (Nieuwenhuijse et al. 2012, Murray et al. 2013). These studies found no late revisions after 10 years. In Nordic countries the CPT stem was used in 6,222 THAs (5,630 with Palacos) from 1995 to 2013. These patients (mean age 73) had a 98.7% 10-year implant survivorship with aseptic loosening as endpoint (Junnila et al. 2016).

For both cement groups in our study, stem retroversion was below 1.8 degrees at 2-year follow-up, which is in accordance with reported retroversion of other similar polished stem types, such as findings of 1.42 degrees retroversion for the Exeter stem (Stryker) (Nieuwenhuijse et al. 2012), 1.6 degrees retroversion of the C-stem (DePuy) (von Schewelov et al. 2014) and 1.58 degrees for C-stem and 1.43 degrees for Exeter stem (Flatoy et al. 2015) at 2 years’ follow-up.

### Clinical evaluation

The clinical evaluation of the slow-curing Hi-Fatigue G bone cement revealed that curing time for Hi-Fatigue G was more than 2 minutes longer as compared with Palacos R + G bone cement. This may seem like a short time, but it is in fact a longer waiting time, where the surgeon needs to apply manual pressure support on the femoral stem in the bone cement during cement curing.

Our patients reached a mean OHS score slightly better than the Danish background population-based OHS score of 40 and better than the threshold (OHS = 40) correspondent to acceptable symptoms after THR (Paulsen et al. 2012, Keurentjes et al. 2014).

### Cement curing

The mixing time and waiting phase in our Palacos R + G group correspond to the in-vitro findings of Dall et al. (2007) who presented mixing time of 50 seconds and waiting phase of 55 seconds. The working phase of 383 seconds and setting time of 76 seconds in the Dall study is, however, a bit lower compared with our in-vivo findings, and could be explained by a higher storage temperature in the Dall study resulting in shorter working time (Kuehn et al. 2005). In our study, the relatively low theater temperature combined with good correlation between storing/theater temperature curing time for Hi-Fatigue G can also explain why Hi-Fatigue G exhibits longer curing time than Palacos R + G.

Slow-curing Hi-Fatigue G bone cement shows better fatigue test in comparison with Palacos R + G bone cement (Tanner 2008). This factor may be important for long-term survival of cemented implants, and short-term (2 years) follow-up RSA in this study revealed only small translations and rotation of CPT stems inserted with Hi-Fatigue G bone cement, which gives positive expectations for long-term survival (Olerud et al. 2014, Meinardi et al. 2016).

### Strengths and weaknesses

The generalizability of the study results translates into elderly (> 70 years) non-osteoporotic hip osteoarthritis patients treated with cemented CPT femoral stem. Yet cemented femoral stems are typically used in older more fragile and often osteoporotic patients, i.e. in the treatment of displaced intracapsular femoral neck fracture by hemiarthroplasty or total hip arthroplasty. Another limitation in this study is a high number of exclusions and non-consenters. The typical reason for declining to participate in the study was poor health and inability to show for several follow-ups, and potentially there is a selection bias towards more fit and mobile elderly patients in the study compared with the typical elderly THA patient.

We expected less stem subsidence with slow-curing Hi-Fatigue G cement because of better bone penetration. However, we found no difference in stem subsidence between cement groups in this study. We did not put markers in the bone cement, and thus we do not know if the measured subsidence took place in the stem-mantle zone or the bone cement junction (Stefansdottir et al. 2004).

In conclusion, this study showed equivalent femoral stem subsidence with Hi-Fatigue G as compared with Palacos R + G bone cement until 2 years’ follow-up. We found 2 minutes longer curing time for Hi-Fatigue G compared with Palacos R + G bone cement. Migrations were similar to other studies of cemented polished stems, and clinical outcomes were above the threshold for acceptable symptoms after THR. Based on this study we expect similar long-term results for fixation of the CPT stem with Hi-Fatigue G and Palacos R + G bone cements.

### Supplementary data

Tables 2–7 are available as supplementary data in the online version of this article, http://dx.doi.org/10.1080/17453674. 2019.1595390

PBJ and MS wrote the manuscript and performed the statistical analyses. ML, KS and MS contributed to planning, interpretation of data, and critical review of the manuscript.

Note: All product names are for identification purposes only, and may be trademarks of their respective owners.

*Acta* thanks Stephan Maximilian Röhrl and Olof Sköldenberg for help with peer review of this study.

## Supplementary data

## Supplementary Material

Supplementary Material

## References

[CIT0001] Arora M, Chan E K S, Gupta S, Diwan A D Polymethylmethacrylate bone cements and additives: a review of the literature. World J Orthop 2013; 4(2): 67–74. 23610754 10.5312/wjo.v4.i2.67 PMC3631954

[CIT0002] Barrack R L, Mulroy R D Jr, Harris W H Improved cementing techniques and femoral component loosening in young patients with hip arthroplasty: a 12-year radiographic review. J Bone Joint Surg Br 1992; 74(3): 385–9. 1587883 10.1302/0301-620X.74B3.1587883

[CIT0003] Dall G F, Simpson P M, Breusch S J In vitro comparison of Refobacin-Palacos R with Refobacin Bone Cement and Palacos R + G. Acta Orthop 2007; 78(3): 404–11. 17611856 10.1080/17453670710013997

[CIT0004] DHR National Report 2016. The Danish Hip Arthroplasty Registry; 2016.

[CIT0005] Flatoy B, Rohrl S M, Rydinge J, Dahl J, Diep L M, Nordsletten L Triple taper stem design shows promising fixation and bone remodelling characteristics: radiostereometric analysis in a randomised controlled trial. Bone Joint J 2015; 97-B(6): 755–61. 10.1302/0301-620X.97B6.34736 26033054

[CIT0006] Glyn-Jones S, Alfaro-Adrian J, Murray D W, Gill H S The influence of surgical approach on cemented stem stability: an RSA study. Clin Orthop Relat Res 2006; 448: 87–91. 16826101 10.1097/01.blo.0000224006.25636.cc

[CIT0007] Hauptfleisch J, Glyn-Jones S, Beard D J, Gill H S, Murray D W The premature failure of the Charnley Elite-Plus stem: a confirmation of RSA predictions. J Bone Joint Surg Br 2006; 88(2): 179–83. 16434520 10.1302/0301-620X.88B2.17055

[CIT0008] ISO International Standard ISO 5833:2002. Implant for surgery: acrylic resin cements. Geneva: ISO; 2002.

[CIT0009] ISO International standard ISO 16087:2013. Implants for surgery: roentgen stereophotogrammetric analysis for the assessment of migration of orthopaedic implants. Geneva: ISO; 2013.

[CIT0010] Junnila M, Laaksonen I, Eskelinen A, Pulkkinen P, Ivar Havelin L, Furnes O, Marie Fenstad A, Pedersen A B, Overgaard S, Karrholm J, Garellick G, Malchau H, Makela K T Implant survival of the most common cemented total hip devices from the Nordic Arthroplasty Register Association database. Acta Orthop 2016; 87(6): 546–53. 27550058 10.1080/17453674.2016.1222804 PMC5119435

[CIT0011] Kaptein B L, Valstar E R, Spoor C W, Stoel B C, Rozing P M Model-based RSA of a femoral hip stem using surface and geometrical shape models. Clin Orthop Relat Res 2006; 448: 92–7. 16826102 10.1097/01.blo.0000224010.04551.14

[CIT0012] Karrholm J, Borssen B, Lowenhielm G, Snorrason F Does early micromotion of femoral stem prostheses matter? 4–7-year stereoradiographic follow-up of 84 cemented prostheses. J Bone Joint Surg Br 1994; 76(6): 912–17. 7983118

[CIT0013] Keurentjes J C, Van Tol F R, Fiocco M, So-Osman C, Onstenk R, Koopman-Van Gemert A W, Poll R G, Nelissen R G Patient acceptable symptom states after total hip or knee replacement at mid-term follow-up: thresholds of the Oxford hip and knee scores. Bone Joint Res 2014; 3(1): 7–13. 24421318 10.1302/2046-3758.31.2000141 PMC3928564

[CIT0014] Kuehn K D, Ege W, Gopp U Acrylic bone cements: composition and properties. Orthop Clin North Am 2005; 36(1): 17–28, v. 15542119 10.1016/j.ocl.2004.06.010

[CIT0015] Meinardi J E, Valstar E R, Van Der Voort P, Kaptein B L, Fiocco M, Nelissen R G Palacos compared to Palamed bone cement in total hip replacement: a randomized controlled trial. Acta Orthop 2016; 87(5): 473–8. 27329869 10.1080/17453674.2016.1199146 PMC5016905

[CIT0016] Murray D W, Gulati A, Gill H S Ten-year RSA-measured migration of the Exeter femoral stem. Bone Joint J 2013; 95-B(5): 605–8. 23632668 10.1302/0301-620X.95B5.31330

[CIT0017] Nelissen R G, Pijls B G, Karrholm J, Malchau H, Nieuwenhuijse M J, Valstar E R RSA and registries: the quest for phased introduction of new implants. J Bone Joint Surg Am 2011; 93(Suppl 3): 62–5. 10.2106/JBJS.K.00907 22262426

[CIT0018] Nieuwenhuijse M J, Valstar E R, Kaptein B L, Nelissen R G The Exeter femoral stem continues to migrate during its first decade after implantation: 10-12 years of follow-up with radiostereometric analysis (RSA). Acta Orthop 2012; 83(2): 129–34. 22401676 10.3109/17453674.2012.672093 PMC3339525

[CIT0019] Olerud F, Olsson C, Flivik G Comparison of Refobacin bone cement and palacos with gentamicin in total hip arthroplasty: an RSA study with two years follow-up. Hip Int 2014; 24(1): 56–62. 24062223 10.5301/hipint.5000088

[CIT0020] Paulsen A, Odgaard A, Overgaard S Translation, cross-cultural adaptation and validation of the Danish version of the Oxford hip score: assessed against generic and disease-specific questionnaires. Bone Joint Res 2012; 1(9): 225–33. 23610695 10.1302/2046-3758.19.2000076 PMC3626210

[CIT0021] Race A, Miller M A, Clarke M T, Mann K A, Higham P A The effect of low-viscosity cement on mantle morphology and femoral stem micromotion: a cadaver model with simulated blood flow. Acta Orthop 2006; 77(4): 607–16. 16929438 10.1080/17453670610012683

[CIT0022] Rey R M Jr, Paiement G D, McGann W M, Jasty M, Harrigan T P, Burke D W, Harris W H A study of intrusion characteristics of low viscosity cement Simplex-P and Palacos cements in a bovine cancellous bone model. Clin Orthop Relat Res 1987; (215): 272–8. 3802646

[CIT0023] Selvik G Roentgen stereophotogrammetry. Acta Orthop Scand 1989; 60(Suppl. 232): 1–51. 2686344

[CIT0024] Stefansdottir A, Franzen H, Johnsson R, Ornstein E, Sundberg M Movement pattern of the Exeter femoral stem: a radiostereometric analysis of 22 primary hip arthroplasties followed for 5 years. Acta Orthop Scand 2004; 75(4): 408–14. 15370583 10.1080/00016470410001169-1

[CIT0025] Stone J J, Rand J A, Chiu E K, Grabowski J J, An K N Cement viscosity affects the bone cement interface in total hip arthroplasty. J Orthop Res 1996; 14(5): 834–7. 8893780 10.1002/jor.1100140523

[CIT0026] Tanner K Fatigue testing of four bone cements for AAP Biomaterials GmbH Co. Test Report. London: Department of Materials, Queen Mary University of London; 2008.

[CIT0027] Valstar E R, Gill R, Ryd L, Flivik G, Borlin N, Karrholm J Guidelines for standardization of radiostereometry (RSA) of implants. Acta Orthop 2005; 76(4): 563–72. 16195075 10.1080/17453670510041574

[CIT0028] van der Voort P, Pijls B G, Nieuwenhuijse M J, Jasper J, Fiocco M, Plevier J W, Middeldorp S, Valstar E R, Nelissen R G Early subsidence of shape-closed hip arthroplasty stems is associated with late revision: a systematic review and meta-analysis of 24 RSA studies and 56 survival studies. Acta Orthop 2015; 86(5): 575–85. 25909455 10.3109/17453674.2015.1043832 PMC4564780

[CIT0029] von Schewelov T, Carlsson A, Sanzen L, Besjakov J Continuous distal migration and internal rotation of the C-stem prosthesis without any adverse clinical effects: an RSA study of 33 primary total hip arthroplasties followed for up to ten years. Bone Joint J 2014; 96-b(5): 604–8. 24788493 10.1302/0301-620X.96B5.33580

[CIT0030] Yates P J, Burston B J, Whitley E, Bannister G C Collarless polished tapered stem: clinical and radiological results at a minimum of ten years’ follow-up. J Bone Joint Surg Br 2008; 90(1): 16–22. 18160493 10.1302/0301-620X.90B1.19546

