# Supplementary material for: Equivalent hip stem fixation by Hi-Fatigue G and Palacos R + G bone cement: a randomized radiostereometric controlled trial of 52 patients with 2 years’ follow-up
Source: Acta Orthop. 2019 Apr 1;90(3):237–42. doi: 10.1080/17453674.2019.1595390 (PMC6534245; doi:10.1080/17453674.2019.1595390)
Supplement: Supplementary Material [file IORT_A_1595390_SM2863.pdf]

Supplementary data

Table 2. Correlation of migration and clinical outcomes

2-year outcome	Subsidence Rho	Subsidence p-value	Retroversion Rho	Retroversion p-value
OHS	–0.19	0.2	–0.09	0.6
Pain, rest	0.15	0.3	–0.04	0.8
Pain, activity	0.08	0.6	–0.01	1.0

Table 3. Precision of RSA

	Translation (mm) X-axis	Translation (mm) Y-axis	Translation (mm) Z-axis	Rotation (°) X-axis	Rotation (°) Y-axis	Rotation (°) Z-axis
Mean diff.	0.01	0.01	0.00	0.02	0.22	–0.03
SD diff.	0.10	0.08	0.15	0.39	1.05	0.16
CR ±	0.19	0.16	0.30	0.77	2.06	0.32
LoA, lower	–0.18	–0.16	–0.30	–0.73	–1.73	–0.34
upper	0.21	0.17	0.30	0.77	2.17	0.30

Mean diff: The systematic difference of RSA.
SD diff: Random variation of RSA.
CR: Coefficient of repeatability (SD*1.96). Indicates the RSA precision for individual recordings.
LoA: Limits of agreement/prediction interval.

Table 4. Migration and clinical outcome

	0 to 1 year Hi–Fatigue (n = 25)	0 to 1 year Palacos (n = 26)	p-value [a]	0 to 2 years Hi–Fatigue (n = 24)	0 to 2 years Palacos (n = 24)	Difference [b]	p-value [a]
Migration (translation), mean (95% CI), mm							
x-axis	0.04 (–0.06 to 0.13)	0.02 (–0.04 to 0.08)	0.7	–0.04 (–0.14 to 0.07)	0.03 (–0.03 to 0.10)	–0.70 (–0.20 to 0.06)	0.4
y-axis	–0.91 (–1.02 to –0.81)	–1.03 (–1.15 to –0.91)	0.2	–1.12 (–1.29 to –0.96)	–1.19 (–1.34 to –1.03)	0.06 (–0.17 to 0.30)	0.7
z-axis	–0.15 (–0.28 to –0.01)	–0.26 (–0.35 to –0.18)	0.1	–0.23 (–0.33 to –0.12)	–0.37 (–0.45 to –0.29)	0.14 (0.00 to 0.28)	0.1
Rotation (°)							
x-axis	–0.18 (–0.44 to 0.09)	–0.09 (–0.29 to 0.11)	0.6	–0.19 (–0.46 to 0.07)	–0.11 (–0.32 to 0.10)	–0.09 (–0.43 to 0.26)	0.6
y-axis	0.90 (0.37 to 1.43)	1.34 (0.94 to 1.85)	0.2	1.14 (0.73 to 1.54)	1.75 (1.27 to 2.24)	–0.62 (–1.26 to 0.03)	0.1
z-axis	0.01 (–0.10 to 0.12)	–0.04 (–0.10 to 0.02)	0.8	–0.14 (–0.27 to 0.00)	–0.09 (–0.16 to –0.03)	–0.62 (–1.26 to 0.03)	0.6
Summed migration, median (CI)							
MTPM (mm) [c]	1.73 (1.14 to 1.87)	1.67 (1.42 to 1.79)	0.9	1.91 (1.52 to 2.07)	1.88 (1.61 to 2.03)	–	0.8
Total translation (mm)	1.01 (0.85 to 1.10)	1.10 (0.95 to 1.18)	0.4	1.21 (1.01 to 1.31)	1.27 (1.09 to 1.36)	–	0.7
Total rotation (°)	1.51 (0.94 to 1.61)	1.58 (0.96 to 1.66)	0.8	1.46 (0.93 to 1.59)	1.92 (1.13 to 2.09)	–	0.2
Clinical outcome, mean (CI)							
Oxford Hip Score	16.2 (11.5 – 20.9)	19.0 (15.5 – 22.4)	0.7	17.0 (11.2 – 22.8)	19.9 (16.3 – 23.4)		1.0
Pain rest (VAS 1–10)	–2.7 (–4.0 to –1.4)	–2.5 (–3.3 to –1.6)	0.4	–2.2 (–3.9 to –0.5)	–2.4 (–3.3 to –1.5)		0.8
Pain activity (VAS 1–10)	–4.3 (–5.7 ot –2.9)	–4.9 (–6.0 to –3.9)	0.6	–4.1 (–5.9 to –2.2)	–4.9 (–5.9 to –3.9)		0.8

[a] Two-sample Wilcoxon rank-sum (Mann–Whitney) test.
[b] Difference in 2-year follow-up by Student's t-test.
[c] MTPM: Maximum total point motion.

Table 5. Postoperative radiographic evaluation according to Barrack et al. (1992)

	Hi-Fatigue (n = 25)	Palacos (n = 26)
Cementation		
A	24	15
B	1	10
C	0	1
D	0	0
Stem position		
Varus	2	2
Neutral	23	24
Valgus	0	0

Table 6. Intraoperative evaluation of bone cements

	Hi-Fatigue (n = 24)	Palacos (n = 27)
Time (minutes) for surgery [a]	83 (55–114)	82 (50–150)
Temperature (°C) in theater [a]	20 (18.3–21)	20(19–21.5)
Humidity (%) in theater [a]	41 (17–72)	41 (19–78)
Temperature (°) in storag [a]	20 (17.5–21)	20(18–21.4)
Humidity (%) in storage [a]	42 (24–72)	50 (26–71)
Mixing of cement (mean min:s, Ci)	1:05 (0:57–1:13)	1:01 (0.53–1:09)
Application of cement begins [b]	2:23 (2:12–2:34)	2:16 (2:06–2:26)
Insertion of femoral stem begins [b]	04:05 (03:54–04:16)	03:49 (03:40–03:58)
Curing of cement [b]	13:43 (13:16–14:11)	11:35 (11:14–11:56)
Were there problems preparing the MixiGun? (yes/no)	0/25	3/24
User-friendliness for preparing MixiGun [c]	6 (2–9)	6 (2–9)
Was the cement easy to mix? (yes/no)	24/1	23/2
Force used for cement mixing [c]	2 (1–7)	2 (2–8)
Was the cement smooth after mixing? (yes/no) 24/0	26/1	
User-friendliness for mixing with MixiGun [c]	3 (2–8)	3 (2–8)
Were there monomer smell problems? [c]	3 (1–8)	3 (1–8)
Was the application ok (surgeon evaluation)? (yes/no)	23/1	27/0
Force used for application of mixing [c]	3 (1–7)	3 (1–7)
Force used for insertion of femoral stem [c]	3 (2–5)	3 (3–8)
Stickiness of the cement at time of application [c]	3 (2–7)	3 (1–7)
Rubberyness of the cement at time of application [c]	3 (1–5)	3 (3–7)
User-friendliness for MixiGun (OR nurse) [c]	5.5 (2–8)	5.5 (2–8)
User-friendliness for MixiGun (surgeon) [c]	7 (2–9)	7 (2–9)

[a] Values are median (range)
[b] Values are mean time in minutes:seconds (CI)
[c] Range from 1 = least to 9 = most

Table 7. Correlation of storage/theater temperature and working times

	Hi-Fatigue		Palacos	
	Rho	p-value	Rho	p-value
Storage temperature				
Mixing	–0.22	0.3	–0.20	0.4
Application of cement begins	–0.14	0.5	0.16	0.4
Application of stem begins	–0.09	0.7	–0.14	0.5
Curing of cement	–0.41	0.1	0.00	1.0
Theater temperature				
Mixing	–0.10	0.7	0.03	0.9
Application of cement begins	–0.13	0.6	0.24	0.3
Application of stem begins	0.20	0.4	–0.07	0.8
Curing of cement	–0.46	0.03	–0.13	0.5
